# Development and validation of a nomogram prediction model for postoperative complications in elderly patients with endometrial cancer

**DOI:** 10.12669/pjms.42.7.14874

**Published:** 2026-07

**Authors:** Yanhong Geng, Qiuju Wang, Jing Xu, Shuqi Guo, Shanshan Liang

**Affiliations:** 1 Yanhong Geng, Department of Obstetrics and Gynecology, Baoding No.1 Central Hospital, Baoding 071000, Hebei, China; 2 Qiuju Wang, Department of Obstetrics and Gynecology, Baoding No.1 Central Hospital, Baoding 071000, Hebei, China; 3 Jing Xu, Department of Obstetrics and Gynecology, Baoding No.1 Central Hospital, Baoding 071000, Hebei, China; 4 Shuqi Guo, Department of Obstetrics and Gynecology, Baoding No.1 Central Hospital, Baoding 071000, Hebei, China; 5 Shanshan Liang, Department of Obstetrics and Gynecology, Baoding No.1 Central Hospital, Baoding 071000, Hebei, China

**Keywords:** Complication, Elderly, Endometrial cancer, Surgery, Predictive model

## Abstract

**Objective:**

To identify the risk factors associated with postoperative complications in elderly patients with endometrial cancer and to develop a nomogram model for individualized risk assessment.

**Methodology:**

This retrospective study included 150 elderly patients who underwent surgical treatment for endometrial cancer at Baoding No.1 Central Hospital between January 2022 to October 2025. Patients were divided into a complication group(n=46) and a non-complication group(n=104) based on the occurrence of postoperative complications. Multivariate logistic regression analysis was conducted to identify risk factors. A nomogram model was constructed and validated accordingly.

**Results:**

Postoperative complications occurred in 30.67%(46/150) of patients, while 69.33%(104/150) experienced no complications. Statistically significant differences were observed between the complication and non-complication groups in age(*t=* 3.021), body mass index(BMI)(*t=* 4.653), presence of diabetes(*χ*²= 6.322), duration of operation(DoO)(*t=* 5.181), and delayed postoperative ambulation (DPA)(*χ*² = 5.771) (all *p<* 0.05). Multivariate analysis identified age (odds ratio[OR] = 3.058), BMI(OR= 2.151), diabetes(OR= 1.865), DoO(OR= 2.677), and DPA(OR= 2.371) as independent risk factors(all *p<* 0.05). A nomogram incorporating these five variables demonstrated excellent predictive performance, with a sensitivity of 89.64%, a specificity of 77.92%, and an area under the receiver operating characteristic curve(AUC) of 0.887(95% confidence interval: 0.833–0.906), indicating high accuracy and clinical utility.

**Conclusion:**

Age, BMI, diabetes, DoO, and DPA are significant risk factors for postoperative complications in elderly patients with endometrial cancer. The constructed nomogram provides a reliable and practical tool for the early identification of high-risk patients and may facilitate the development of targeted preventive strategies in clinical practice.

## INTRODUCTION

Endometrial cancer is one of the most common malignant tumors affecting women, with its incidence and mortality rates rising continuously. Several factors, such as obesity, socioeconomic status, race, and age, are closely associated with the risk and prognosis of endometrial cancer. With the majority of patients being between 65 and 75 years of age, the advanced age of this patient population poses considerable challenges for treatment management.^1^,^2^ Surgical intervention remains the mainstay of treatment for endometrial cancer, often supplemented by radiotherapy and chemotherapy to reduce the risk of recurrence. Surgical options include open surgery and minimally invasive procedures like laparoscopy. For select patients with fertility preservation needs, disease progression may be temporarily managed with oral progestins, followed by definitive surgical treatment.^3^

Despite its clinical benefits, surgical treatment carries the risk of postoperative complications, including infection, hemorrhage, wound dehiscence, injury to adjacent organs, and intestinal obstruction. These complications can significantly hinder postoperative recovery and adversely impact patient health. Their occurrence is influenced by diverse factors, such as cancer stage, comorbidities, obesity, intraoperative blood loss (IBL), and duration of operation(DoO).^4^ Currently, there is a lack of robust evaluation tools specifically designed to predict postoperative complications in elderly patients with endometrial cancer. To achieve timely intervention and improve the clinical outcomes of elderly patients with endometrial cancer, it is of critical importance to identify risk factors for postoperative complications in this vulnerable population.

The development of a nomogram prediction model could offer a practical, visual method for assessing the individualized risk of postoperative complications in elderly patients with endometrial cancer. Such a tool would aid in early risk stratification and inform clinical decision-making to mitigate the likelihood of adverse surgical outcomes. This study aimed to identify the risk factors associated with postoperative complications in elderly patients with endometrial cancer and to construct and validate a nomogram prediction model.

## METHODOLOGY

This was a retrospective study. A total of 150 elderly patients who underwent surgical treatment for endometrial cancer at Baoding No.1 Central Hospital between January 2022 to October 2025 were retrospectively included in this study. Patient data including demographic data, main clinical features were retrieved from electronic medical record systems. Patients were divided into a complication group(n=46) and a non-complication group(n=104) based on the occurrence of postoperative complications.

### Ethical Approval:

The study was approved by the Institutional Ethics Committee of Baoding No.1 Central Hospital(No.:[2025]162; Date: August 20, 2025), and written informed consent was obtained from all participants.

### Inclusion criteria:

- Diagnosis confirmed according to established clinical criteria for endometrial cancer.^5^
- Age ≥60 years.
- Receiver of surgical treatment.
- Complete clinical data available.
- No prior treatment before hospital admission.
- Signed informed consent.

### Exclusion criteria:

- Concomitant malignancies.
- Dysfunction of other major organs.
- Coagulation disorders.
- Comorbid psychiatric conditions.
- Poor treatment compliance.

### Types of Postoperative Complications:

Of the listed complications were categorized as the non-complication group. All the procedure Postoperative complications in elderly patients with endometrial cancer were documented, including nausea and vomiting, bleeding, lower extremity venous thrombosis, subcutaneous emphysema, visceral injury, infection, shoulder pain, and throat pain. Patients who experienced one or more of these complications were assigned to the complication group; those without any s in both groups done by the same group of doctors. The maximum follow-up time for patients in both groups was six months. And case data collection ceased in October 2025.

### Data Collection:

Clinical data were extracted from the hospital information system, including age, body mass index (BMI), comorbidities (*e.g*., anemia, hypertension, diabetes, hyperlipidemia), surgical approach (laparoscopic or open surgery), lymph node metastasis, pathological stage,^6^ DoO, duration of anesthesia (DoA), IBL, blood transfusion, and delayed postoperative ambulation (DPA, defined as being bedridden for more than 48 hours after surgery).

### Statistical analysis:

All statistical analyses were performed using SPSS 26.0. Continuous variables were expressed as mean ± standard deviation (), and categorical variables were presented as frequency and percentage (*n*[%]). Group comparisons were conducted using the *t*-test for continuous variables and the chi-square (*χ*²) test for categorical variables. Multivariate logistic regression analysis was used to identify independent risk factors for postoperative complications in elderly patients with endometrial cancer. A nomogram prediction model was developed using R version 3.5.3 and associated packages. The discriminative ability of the nomogram was evaluated using the receiver operating characteristic(ROC) curve and the area under the curve (AUC). Model calibration was assessed using the Hosmer-Lemeshow goodness-of-fit test. The clinical utility of the model was further evaluated through decision curve analysis (DCA). A *P*-value of less than 0.05 was considered statistically significant.

## RESULTS

Among the 150 elderly patients who underwent surgical treatment for endometrial cancer, postoperative complications occurred in 46 patients, accounting for 30.67%, while the remaining 104 patients (69.33%) experienced no postoperative complications (Table-I).

**Table-I:** Incidence of postoperative complications in elderly patients with endometrial cancer.

Complication	Complication group (n = 46)
Nausea and vomiting	13
Bleeding	6
Lower extremity venous thrombosis	3
Subcutaneous emphysema	6
Visceral injury	1
Infection	6
Shoulder pain	11
Throat pain	4

A comparison of clinical variables between the complication group and the non-complication group is shown in Table-II. The two groups exhibit no statistically significant differences in anemia(*χ*² = 2.696), hypertension(*χ*² = 1.513), hyperlipidemia(*χ*² = 3.191), surgical approach(*χ*² = 2.634), lymph node metastasis(*χ*² = 2.719), pathological stage(*χ*² = 2.250), DoA(*t =* 1.852), IBL(*t =* 1.727), or blood transfusion(*χ*² = 1.272), with all P-values greater than 0.05. In contrast, statistically significant differences were observed in age(*t =* 3.021), BMI(*t =* 4.653), diabetes(*χ*² = 6.322), DoO(*t =* 5.181), and DPA(*χ*² = 5.771) (*p<* 0.05, respectively). Patients in the complication group had significantly higher age, BMI, longer DoO, and higher proportions of diabetes and DPA compared with the non-complication group.

**Table-II:** Comparison of clinical characteristics between the complication and non-complication groups.

Variable	n	Complication group (n = 46)	Non-complication group (n = 104)	χ²/t	P-value
Age (years)		72.65±6.38	69.33±6.13	3.021	0.003
BMI (kg/m²)		26.15±1.84	24.73±1.67	4.653	0.000
Anemia				2.696	0.101
Present	36	15(32.61)	21(20.19)		
Absent	114	31(67.39)	83(79.81)		
Hypertension				1.513	0.219
Present	67	24(52.17)	43(41.35)		
Absent	73	22(47.83)	61(58.65)		
Diabetes				6.322	0.012
Present	47	21(45.65)	26(25.00)		
Absent	103	25(54.35)	78(75.00)		
Hyperlipemia				3.191	0.074
Present	35	15(32.61)	20(19.23)		
Absent	115	31(67.39)	84(80.77)		
Surgical approach				2.634	0.105
Laparoscopic	105	28(60.87)	77(74.04)		
Open	45	18(39.13)	27(25.96)		
Lymph node metastasis				2.719	0.099
Present	57	22(47.83)	35(33.65)		
Absent	93	24(52.17)	69(66.35)		
Pathological stage				2.250	0.134
I–II	101	27(58.70)	74(71.15)		
III	49	19(41.30)	30(28.85)		
DoO (min)		176.93±43.73	141.56±36.06	5.181	0.000
DoA (h)		2.85±0.59	2.67±0.53	1.852	0.066
IBL (mL)		309.02±82.72	286.67±68.43	1.727	0.086
Blood transfusion				1.272	0.259
Yes	22	9(19.57)	13(12.50)		
No	128	37(80.43)	91(87.50)		
DPA				5.771	0.016
Yes	31	15(32.61)	16(15.38)		
No	119	31(67.39)	88(84.62)		

A multivariate logistic regression analysis was performed to identify independent risk factors for postoperative complications in elderly patients with endometrial cancer. The occurrence of complications (absent*=* 0, present *=* 1) was set as the dependent variable. Independent variables included age (continuous), BMI (continuous), diabetes (absent= 0, present*=* 1), DoO (continuous), and DPA (no = 0, yes = 1). As shown in Table-III, age (odds ratio [OR] = 3.058), BMI (OR = 2.151), diabetes (OR = 1.865), DoO (OR = 2.677), and DPA (OR = 2.371) are significant risk factors for the development of postoperative complications (all *p<* 0.05).

**Table-III:** Risk factors for postoperative complications in elderly patients with endometrial cancer.

Variable	β	SE	Wald χ²	P-value	OR	95% CI
Age	1.118	0.473	5.584	0.018	3.058	1.210–7.728
BMI	0.766	0.345	4.929	0.026	2.151	1.094–4.230
Diabetes	0.623	0.308	4.095	0.043	1.865	1.020–3.411
DoO	0.985	0.376	6.859	0.009	2.677	1.281–5.594
DPA	0.863	0.352	6.015	0.014	2.371	1.189–4.727
Constant	-64.127	0.489	29.723	<0.001	-	-

**Fig.1:**
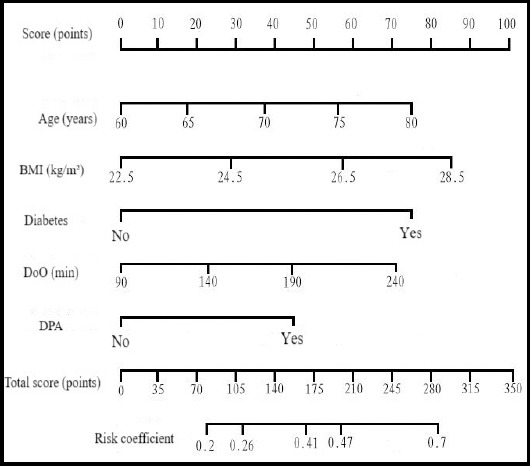
Nomogram model for predicting the risk of postoperative complications in elderly patients with endometrial cancer.

A nomogram model was constructed based on the five identified independent risk factors, namely age, BMI, diabetes, DoO, and DPA, to visually predict the risk of postoperative complications in elderly patients with endometrial cancer. Fig.1. The total risk score is obtained by summing the individual scores of each sub-variable in the model. A higher total score corresponds to a higher predicted risk of complications, while a lower score indicates a reduced risk.

**Fig.2:**
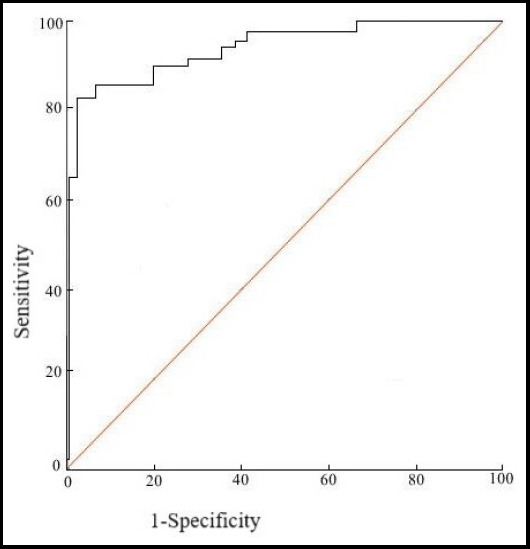
Predictive performance of the nomogram model for postoperative complications in elderly patients with endometrial cancer.

**Fig.3:**
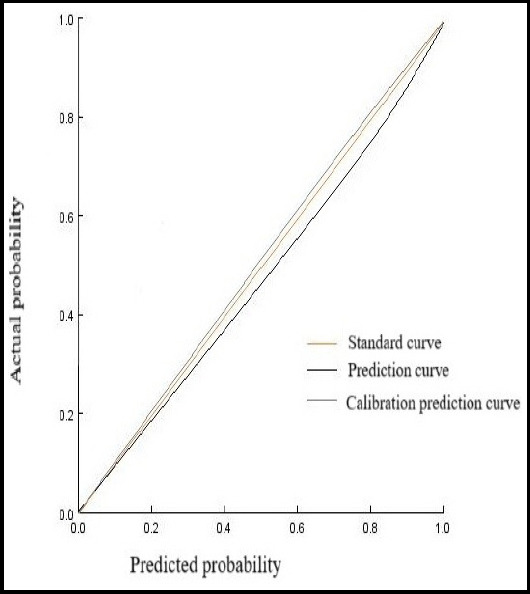
Calibration curve of the nomogram model for postoperative complication risk in elderly patients with endometrial cancer.

The predictive performance of the nomogram model for assessing the risk of postoperative complications in elderly patients with endometrial cancer is displayed in Fig2. The model demonstrated a sensitivity of 89.64%, specificity of 77.92%, and an AUC of 0.887 (95% confidence interval [95% CI]: 0.833–0.906), indicating excellent discriminatory ability. The Hosmer-Lemeshow goodness-of-fit test showed no statistically significant difference between the predicted and observed outcomes (*χ*² = 2.874, *p=* 0.143), suggesting good calibration of the model. Fig.3.

**Fig.4:**
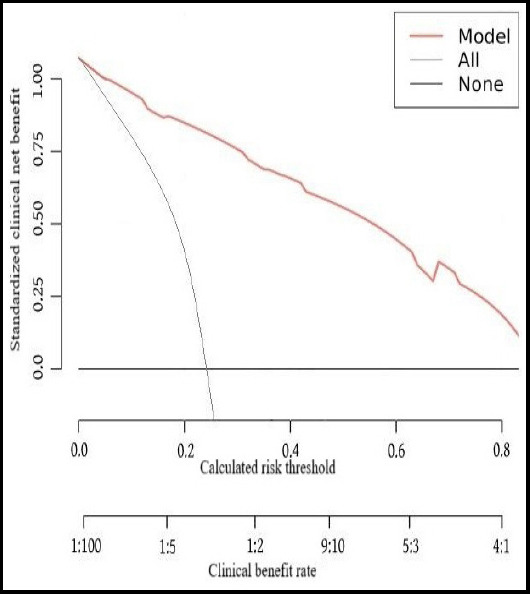
DCA of the nomogram model for assessing the risk of postoperative complications in elderly patients with endometrial cancer.

Fig.4 presents the DCA, which evaluates the clinical utility of the nomogram, and the results indicate that the model has good clinical applicability in assessing the risk of postoperative complications in elderly patients with endometrial cancer.

## DISCUSSION

In the present study, 46 out of 150 elderly patients who underwent surgical treatment for endometrial cancer developed postoperative complications, accounting for 30.67% of the cohort. This highlights the necessity of developing a risk prediction model for postoperative complications in elderly patients with endometrial cancer. Endometrial cancer primarily affects postmenopausal women, with the majority of patients being elderly. Most cases are diagnosed at an early stage, and the prognosis is generally favorable. Clinically, surgical interventions such as hysterectomy are commonly used for treatment.^5^ However, in elderly patients, declining physiological function and the frequent presence of comorbidities like hypertension increase the complexity of treatment and the risk of developing postoperative complications such as cardiovascular events and bleeding.^7^ Previous studies have shown that perioperative management can reduce the risk of complications in patients with endometrial cancer.^8^

This study revealed statistically significant differences in age, BMI, presence of diabetes, DoO, and DPA between patients who developed postoperative complications and those who did not. Patients in the complication group had higher values or incidence rates for these variables compared with those in the non-complication group. Age has been shown to significantly impact surgical outcomes in patients with endometrial cancer. As age increases, immune function tends to decline, postoperative recovery becomes slower, and the risks of complications, prolonged hospitalization, and mortality increase. Compared with younger patients, elderly individuals have a significantly higher likelihood of developing postoperative complications such as thromboembolism, wound infection, and postoperative bleeding.^9^ Pin S *et al*.^10^ reported that advanced age is a significant risk factor for postoperative venous thromboembolism (VTE) in those with cancer. The risk of thrombotic events increases with age, highlighting the importance of implementing preventive measures in the postoperative period to reduce VTE incidence. Obesity has also been identified as a well-established risk factor for the development of endometrial cancer and is associated with adverse surgical outcomes. Excess adiposity can increase the risk of postoperative complications.^11^

In a study by Schipa C *et al*.^12^, both age and BMI were found to be significant predictors of postoperative complications in patients with endometrial cancer. One possible explanation is that obese patients may have larger tumors, require longer DoO, and experience greater IBL, all of which contribute to an elevated risk of complications.^13^ Other studies have demonstrated that obese patients with endometrial cancer tend to have a greater number of lymph nodes excised and that BMI is positively correlated with DoO. This may be due to reduced surgical visibility and increased technical difficulty during the procedure in obese patients compared with those with normal body weight, which in turn prolongs DoO.^14^

Furthermore, an elevated BMI has been associated with poorer survival outcomes in patients with endometrial cancer who develop postoperative complications.^15^ Surgical site infection (SSI) is one of the most common postoperative complications in patients with endometrial cancer. Yang R *et al*.^16^ reported that patients with elevated postoperative blood glucose levels are at significantly higher risk of developing SSIs compared with those with lower glucose levels. This may be explained by the fact that diabetic patients often have elevated glycated hemoglobin levels, which impair immune function. Consequently, patients with diabetes are more susceptible to infections during and after surgery. Preoperative glycemic control is therefore essential to reduce the risk of postoperative infection in diabetic patients with endometrial cancer.^17^ Consistent with the findings of the present study, prolonged DoO has been identified as an independent risk factor for postoperative complications in patients with endometrial cancer. Longer DoO is associated with extended tissue exposure and an increased likelihood of bacterial contamination, thereby elevating the risk of SSIs, particularly at the incision site.^18^

Moreover, postoperative immobility has been shown to adversely affect gastrointestinal and cardiovascular function. Early postoperative ambulation, in combination with prophylactic anticoagulation, can significantly reduce the risk of deep vein thrombosis and pulmonary embolism. In addition, early mobilization can accelerate postoperative recovery, shorten hospital stays, and reduce overall healthcare costs.^19^,^20^

### Limitations

The sample size is small, not all potential risk factors were included in the model, and future research should explore additional variables that may influence the occurrence of postoperative complications in this patient population.

## CONCLUSIONS

This study identified age, BMI, diabetes, DoO, and DPA as independent risk factors for postoperative complications in elderly patients with endometrial cancer. Preoperative screening and identification of these risk factors are essential to minimizing the risk of postoperative complications in this vulnerable population. Individualized treatment regimens should be developed accordingly to reduce the incidence of postoperative complications. Based on these risk factors, a nomogram model was constructed to predict the likelihood of postoperative complications in patients with endometrial cancer. The model demonstrated excellent predictive performance, with a sensitivity of 89.64%, specificity of 77.92%, and an AUC of 0.887, indicating high accuracy and clinical utility worth of broader application.

### Authors’ Contributions:

**YG** and **JX:** Conceived and designed the study, and are responsible and accountable for the accuracy or integrity of the work.

**SG** and **SL:** Collected the data and performed the analysis.

**QW:** Was involved in the writing of the manuscript and is responsible for the integrity of the study.

All authors have read and approved the final manuscript.
